# Obesity as a Risk Factor of Severe Outcome of COVID-19: A Pair-Matched 1:2 Case–Control Study

**DOI:** 10.3390/jcm12124055

**Published:** 2023-06-15

**Authors:** Antonio Russo, Mariantonietta Pisaturo, Verdiana Zollo, Salvatore Martini, Paolo Maggi, Fabio Giuliano Numis, Ivan Gentile, Nadia Sangiovanni, Anna Maria Rossomando, Vincenzo Bianco, Giosuele Calabria, Raffaella Pisapia, Alessio Vinicio Codella, Alfonso Masullo, Elio Manzillo, Grazia Russo, Roberto Parrella, Giuseppina Dell’Aquila, Michele Gambardella, Antonio Ponticiello, Lorenzo Onorato, Nicola Coppola

**Affiliations:** 1 Infectious Diseases Unit, Department of Mental Health and Public Medicine, University of Campania “L. Vanvitelli”, 80138 Napoli, Italy; antonio.russo2@unicampania.it (A.R.); mariantonietta.pisaturo@unicampania.it (M.P.); verdianazollo@gmail.com (V.Z.); salvatoremartini76@gmail.com (S.M.); lorenzoonorato@libero.it (L.O.); 2 Infectious Diseases Unit, A.O. S Anna e S Sebastiano Caserta, 81100 Caserta, Italy; paolo.maggi@unicampania.it; 3 Emergency Unit, P.O. Santa Maria delle Grazie, 80078 Pozzuoli, Italy; fabiogiuliano.numis@aslnapoli2nord.it; 4 Infectious Disease Unit, Department of Clinical Medicine and Surgery, University Federico II, 80131 Naples, Italy; ivan.gentile@unina.it; 5 UOC Systemic and Immunosuppressed Infections, Azienda Ospedaliera di Rilievo Nazionale dei Colli, P.O. Cotugno, 80131 Naples, Italy; nadia.sangiovanni@alice.it; 6 IV Infectious Diseases Unit and Gender Medicine, Azienda Ospedaliera di Rilievo Nazionale dei Coli, P.O. Cotugno, 80131 Naples, Italy; annamaria.russomando@gmail.com; 7 Hepatic Infectious Diseases Unit, Azienda Ospedaliera di Rilievo Nazionale dei Colli, P.O. Cotugno, 80131 Naples, Italy; bianco.vincenzo@yahoo.it; 8 IX Infectious Diseases Unit, Azienda Ospedaliera di Rilievo Nazionale dei Colli, P.O. Cotugno, 80131 Naples, Italy; g.calabria@tin.it; 9 First Infectious Diseases Unit, Azienda Ospedaliera di Rilievo Nazionale dei Coli, P.O. Cotugno, 80131 Naples, Italy; raffipisapia@gmail.com; 10 Infectious Diseases Unit, A.O. San Pio, PO Rummo, 82100 Benevento, Italy; alessioviniciocodella@gmail.com; 11 Infectious Diseases Unit, A.O. San Giovanni di Dio e Ruggi D’Aragona, 84131 Salerno, Italy; al.masullo@alice.it; 12 VIII Infectious Diseases Unit, Azienda Ospedaliera di Rilievo Nazionale dei Coli, P.O. Cotugno, 80131 Naples, Italy; manzillo@libero.it; 13 Infectious Diseases Unit, Ospedale Maria S.S. Addolorata di Eboli, 84025 Eboli, Italy; gr.russo@aslsalerno.it; 14 Respiratory Infectious Diseases Unit, Azienda Ospedaliera di Rilievo Nazionale dei Colli, P.O. Cotugno, 80131 Naples, Italy; roberto.parrella@ospedalideicolli.it; 15 Infectious Diseases Unit, A.O., 83100 Avellino, Italy; dellaquilagiuseppina@libero.it; 16 Infectious Diseases Unit, P.O. S. Luca, 84078 Vallo della Lucania, Italy; gambardella1960@gmail.com; 17 Pneumology Unit and Respiratory Pathophysiology, Azienda Ospedaliera di Rilievo Nazionale Sant’ Anna and San Sebastiano, 81100 Caserta, Italy; antonio.ponticiello@unina.it

**Keywords:** obesity, COVID-19, SARS-CoV-2 infection, severity of disease, mortality

## Abstract

Background and aim. The nature of the association between obesity and poor prognosis of COVID-19 without the evaluation of other co-pathologies associated has not yet been clearly evaluated. The aim of the present pair-matched case–control study was to investigate the outcome of patients with SARS-CoV-2 infection in obese and non-obese patients matched considering gender, age, number of comorbidities, and Charlson Comorbidity Index. Methods. All the adults hospitalized for SARS-CoV-2 infection and with BMI ≥ 30 kg/m^2^ were included (Cases). For each Case, two patients with BMI < 30 kg/m^2^ pair matched for gender, age (±5 years), number of comorbidities (excluding obesity), and Charlson Comorbidity Index (±1) were enrolled (Controls). Results. Of the 1282 patients with SARS-CoV-2 infection followed during the study period, 141 patients with obesity and 282 patients without were enrolled in the case and control groups, respectively. Considering matching variables, there was no statistical difference between the two groups. Patients in the Control group developed more frequently a mild–moderate disease (67% vs. 46.1%, respectively), whereas obese patients were more prone to need intensive care treatment (41.8% vs. 26.6%, respectively; *p* = 0.001). Moreover, the prevalence of death during hospitalization was higher in the Case group than in the Control group (12.1% vs. 6.4%, *p* = 0.046). Discussion. We confirmed an association between obesity and severe outcome of patients with COVID-19, also considering other factors associated with a severe outcome of COVID-19. Thus, in the case of SARS-CoV-2 infection, the subjects with BMI ≥ 30 kg/m^2^ should be evaluated for early antiviral treatment to avoid the development of a severe course.

## 1. Introduction

In 2019, an emerging viral infection caused by a novel coronavirus named SARS-CoV-2 was reported in Wuhan (China). It rapidly spread worldwide, and on 12 March 2020, the World Health Organization (WHO) declared it a pandemic. Italy was the first European country hit by the infection, with the first autochthonous cases registered in Lombardy, a region in the north of the country. By the beginning of March 2020, there were more than 609,247,113 cases and over 6,503,894 deaths worldwide, with these numbers continuing to increase [1].

Coronavirus Infectious Disease-19 (COVID-19) is a multisystemic disorder characterized by a variety of symptoms ranging from non-severe to severe presentation [2]. Both clinical and laboratory parameters have been associated with a non-favorable outcome, such as older age, male sex, and several chronic diseases, including diabetes, hypertension, cardiovascular and renal disease, oncohematological disorders, and dementia [3,4,5,6]. Obesity is a chronic disease characterized by abnormal or excessive fat accumulation; this condition, defined as a body mass index (BMI) > 30 kg/m^2^ according to the WHO, leads to an increased risk of developing several chronic diseases, such as diabetes; cardiovascular diseases (CVDs); depression; non-alcoholic fatty liver disease; several infections, both viral and bacterial; colon diverticulosis; dysbiosis [7]; cancer; and it has also been associated with a significant reduction in life expectancy. Moreover, some studies showed that the risk of cardiovascular disease increased with the increase in BMI class (Class I, BMI 30–34.9 kg/m^2^; Class II, 35–39.9 kg/m^2^; Class III, >40 kg/m^2^ and waist circumference) [8,9,10]. During the past 50 years, obesity has become an increasing condition all over the world. The Global Burden of Disease (GDB) reported that one-third of the world population is currently classified as overweight or obese [11], and according to the State of Food Security and Nutrition in the World, obesity reached a global adult obesity rate of 13.2% in 2019 [12]. The Italian scenario is very similar to the rest of the world; in 2021, 46.2% of the Italian population was characterized by higher body weight, with a prevalence of overweight of 34.2% and obesity of 12% [13].

A condition of increased adipose tissue, especially visceral fat, predisposes to increased morbidity and mortality in patients with SARS-CoV-2 infection, as was also reported for influenza A [14,15]. However, obese subjects more frequently have other chronic conditions, for example, cardiovascular (CVD), liver or kidney disease, and arterial hypertension, which are all associated with a more severe clinical presentation of COVID-19. However, the nature of the association between obesity and poor prognosis of COVID-19 without the evaluation of other associated co-pathologies has not yet been clearly evaluated.

The aim of the present pair-matched case–control study was to investigate the outcome of patients with SARS-CoV-2 infection and obesity, compared with patients with a body mass index < 30 kg/m^2^, but with the same sex, similar age, presence of comorbidities, and Charlson Comorbidity Index, to assess the impact on COVID-19 severity and mortality.

## 2. Materials and Methods

### 2.1. Study Design and Setting

We performed a multicenter, observational, 1:2 matched case–control study involving nine COVID-19 units in eight cities in the Campania region in southern Italy: Naples, Caserta, Salerno, Benevento, Avellino, Pozzuoli, Eboli, and Vallo della Lucania. The patients were adults (≥18 years), hospitalized with a diagnosis of SARS-CoV-2 infection confirmed by a positive reverse transcriptase–polymerase chain reaction (RT-PCR) on a naso-oropharyngeal swab. The study period was from 28 February 2020 to 31 May 2021. The Campania COVID-19 cohort was an observational retrospective, prospective cohort in which all the infectious diseases units and structures that have faced the COVID-19 pandemic in Campania, a region in Southern Italy, have participated and participate. All patients admitted to one of the identified hospital units and confirmed positive to a RT-PCR for SARS-CoV-2 on a naso-oropharyngeal swab were eligible to be enrolled. Inclusion criteria were adults (>18 years old); accept and sign informed consent. Exclusion criteria were patients less than 18 years old; refused the informed consent. All patients were followed up since discharge for hospital or death during hospitalization. The database created for the CoviCamp cohort included all demographic, clinical, and laboratory data relevant for the aim. No study protocol or guidelines regarding the criteria of hospitalization were shared among the centers involved in the study, and the patients were hospitalized following the decision of physicians of each center. The details of the Campania COVID-19 cohort (CoViCamp cohort) were reported in previous studies [4,5,16,17,18].

For the present study, all patients with BMI ≥ 30 kg/m^2^ in the CoViCamp cohort were enrolled as Cases (Case group). For each Case, 2 patients with BMI < 30 kg/m^2^, pair matched for gender, age (±5 years), number of comorbidities (excluding obesity), and Charlson Comorbidity Index (CCI, ± 1), were chosen from the CoviCamp cohort (Control group). Exclusion criteria included minority age, and lack of clinical data and/or informed consent.

The study was approved by the Ethics Committee of the University of Campania L. Vanvitelli, Naples (No. 10877/2020). All procedures performed in this study were in accordance with the ethical standards of the institutional and/or national research committee and with the 1964 Helsinki Declaration and its later amendments or comparable ethical standards. Informed consent was obtained from all participants included in the study. This study was reported following the STROBE recommendations for an observational study (Supplementary Table S1).

### 2.2. Data Collection

All demographic, clinical, and laboratory details of both Cases and Controls were collected in a database. From this database, we extrapolated the data. The variable included in this study were number of patients with obesity (423 valid values), gender (423 valid values), number of patients with arterial hypertension (423 valid values), age (423 valid values), days from admission to discharge (422 valid values), number of comorbidity for each patients (423 valid values), Charlson Comorbidity Index (CCI) (417 valid values), number of patients with cardiovascular disease (423 valid values), number of patients with Chronic Obstructive Pulmonary Disease (COPD) (423 valid values), number of patients with chronic kidney disease (CKD) (423 valid values), number of patients with active cancer (423 valid values), number of patients with hepatopathy (421 valid values), number of patients with diabetes (423 valid values), number of patients with dementia (421 valid values), number of patients with fever (420 valid values), number of patients with dyspnea (419 valid values), number of patients with asthenia (418 valid values), number of patients with ageusia/dysgeusia (418 valid values), number of patients with anosmia/hyposmia (418 valid values), number of patients with diarrhea (418 valid values), number of patients with skin lesion (413 valid values), patients dead during hospitalization (423 valid values), patients with severe outcome or dead during hospitalization (423 valid values), white blood cell count at admission (WBC) (405 valid value), International Normalized ratio (INR) value at admission (376 valid value), lactate dehydrogenase (LDH) value at admission (377 valid value), creatinine value at admission (393 valid values), creatine phosphokinase (CPK) value at admission (274 valid values) aspartate aminotransferase (AST) value at admission (396 valid values), alanine aminotransferase (ALT) value at admission (383 valid values), total bilirubin value at admission (370 valid values), and PaO2/FiO2 (P/F) value at admission (378 valid values).

### 2.3. Definitions

The microbiological diagnosis of SARS-CoV-2 infection was defined as a positive RT-PCR test on a naso-oropharyngeal swab. Viral RNA was extracted from the naso-oropharyngeal swab with QIAamp Viral RNA Kits (Qiagen GmbH, Hilden, Germany); the detection of SARS-CoV-2 was performed by RT-PCR test using Bosphore^®^ Novel Coronavirus (Anatolia Diagnostics and Biotechnology Products Inc., İstanbul, Turkey) Detection Kit V3, by primers designed on three viral regions: E, ORF1ab, and N regions. Variables included in this study and relative numbers are reported in the Supplementary Data. Patients who had BMI ≥ 30 kg/m^2^ were defined as obese in our database, and patients with BMI < 30 kg/m^2^ were defined as non-obese in our database.

Patients were followed until SARS-CoV-2-RNA negativity at naso-oropharyngeal swab or discharged from hospital or death. We defined patients with a mild, moderate, or severe outcome according to the clinical presentation of COVID-19. Precisely, patients with a mild outcome did not need oxygen (O_2_) therapy and/or had a MEWS score below 3 points. Patients with a moderate outcome required non-invasive O_2_ therapy and/or had a MEWS score equal to or above 3 points (≥3). Lastly, patients with a severe outcome needed management in an intensive care unit (ICU) and/or mechanical ventilation (invasive or not invasive); in this definition, we also included patients who died during hospitalization. 

### 2.4. Statistical Analysis

For the descriptive analysis, categorical variables were presented as absolute numbers and their relative frequencies. Continuous variables were summarized as mean and standard deviation or as median and first and third interquartile value (Q1–Q3). We performed a comparison of patients with BMI ≥ 30 kg/m^2^ and with BMI < 30 kg/m^2^ using the Pearson chi-square or Fisher’s exact test for categorical variables and Student’s t- or Mann–Whitney tests for continuous variables. Student’s t-test was used for parametric variables and the Mann–Whitney test for non-parametric variables. To evaluate the distribution of continuous variables, we used the Shapiro–Wilk test. To estimate the survival function, we used log-rank and the Kaplan–Meier curve for a visual representation. A value of *p* < 0.05 was considered statistically significant. Analyses were performed using STATA [19].

## 3. Results

During the study period, 2054 patients with SARS-CoV-2 infection were enrolled in the CoViCamp cohort. For 1282 patients, the status of obesity was reported, and they were enrolled in the present study. No difference in the demographic, clinical, or biochemical data was observed between the 282 patients for whom the datum of obesity was available and for the 772 without the obesity data. Only 18 of 1282 (1.4%) patients had been vaccinated against SARS-CoV-2 with a full schedule (two doses).

**Figure 1:**
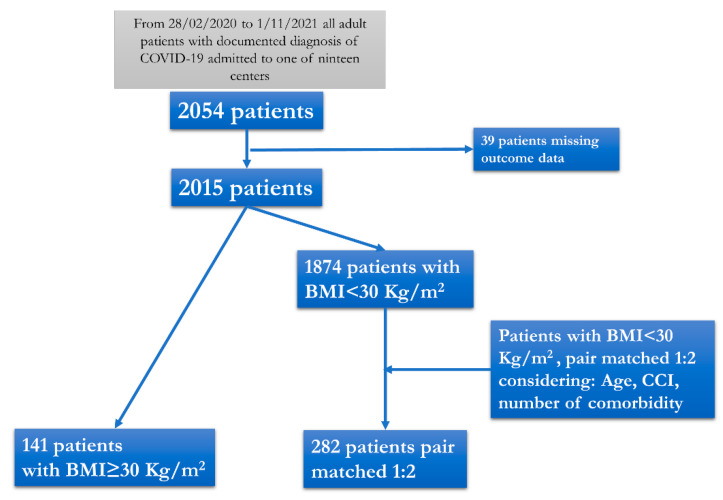
Flow chart of the patients included in the study.

Of the 1282 patients enrolled, 141 (11%) presented at admission with BMI ≥ 30 kg/m^2^ and were included in the Case group, while 282 patients, pair matched for sex, age, CCI, and number of co-pathologies, but with BMI < 30 kg/m^2^, were included in the Control group (Figure 1).

Table 1 shows the demographic and clinical characteristics of the patients in both the Case and Control groups. Most patients were males (67.4%) in both the Case and Control groups, with a mean age of 58 (Q1–Q3 48–69) (Table 1). Obese patients more frequently showed arterial hypertension compared to the Control group (61% vs. 49.6%, *p* = 0.027) (Table 1); instead, no difference between the Cases and Controls was observed in the prevalence of other chronic diseases, such as CVDs, diabetes, chronic liver, and kidney disease (Table 1).

Table 2 compares the clinical and laboratory parameters of the patients in the Case and Control groups. The prevalence of pneumonia was very high in both groups (97.9% and 93.6%, respectively). Additionally, the clinical presentation at admission or in the last days before hospitalization was similar in the Cases and the Controls (Table 2), except for diarrhea, which was more frequent in the Case group (7.2% vs. 2.9%, *p* = 0.038), and asthenia, more frequent in the Control group (28.6% vs. 16.7%, *p* = 0.01) (Table 2). Moreover, the patients in the Case group showed at admission a lower value of PaO2/FiO2 ratio (P/F) (median 194 (Q1–Q3 119.5–300) vs. 232 (Q1–Q3 156–329), respectively; *p =* 0.014) (Table 2). Between the two groups, no statistical differences were observed at admission regarding laboratory parameters, except for blood creatinine level, which was more elevated in the obese group (median 0.9 mg/dL (Q1–Q3 0.8–1.1) vs. 0.84 mg/dL (Q1–Q3 0.7–1.025), respectively, *p* = 0.050) (Table 2).

**Figure 2:**
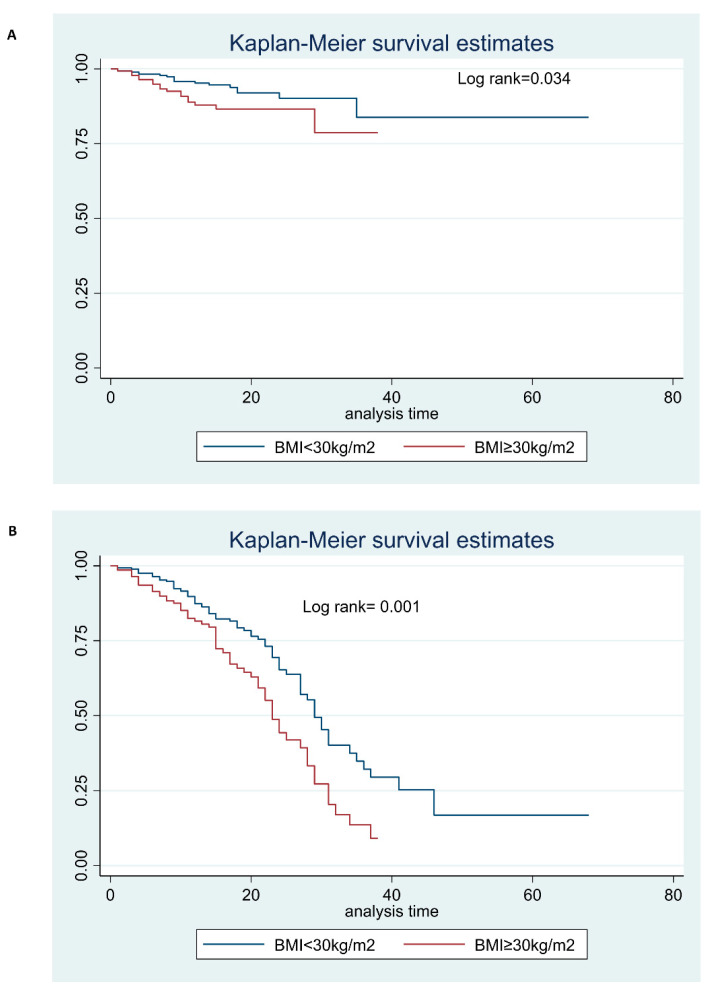
(**A**) Survival curve estimate of patients stratified by BMI during hospitalization; (**B**) Survival curve estimate of ICU care of patients stratified by BMI during hospitalization.

Table 3 shows the differences in the clinical outcome of the patients in the Case and Control groups. Thirty-five patients (8.3%) died during the hospitalization, all for respiratory failure due to COVID-19 pneumonia. Compared to the patients in the Case group, those in the Control group more frequently developed a mild–moderate disease (67% vs. 46.1%, respectively, *p* = 0.0001), whereas obese patients were more prone to be in need of intensive care treatment (41.8% vs. 26.6%, respectively; *p* = 0.001) (Table 3) (Figure 2A). Moreover, the prevalence of death during hospitalization was higher in the Case group than in the Control group (12.1% vs. 6.4%, *p* = 0.046) (Table 3) (Figure 2B). In conclusion, a severe outcome of COVID-19, with or without death during hospitalization was observed in 76 (53.9%) Cases and in 93 (33%, *p* < 0.0001) Controls.

## 4. Discussion

Obesity is one of the most important clinical conditions worldwide. The incidence of obesity in the general population is high and on the increase; worldwide, the prevalence rate for being overweight or obese between 1980 and 2013 increased to 27.5% for adults and 47.1% for children, i.e., a total of 2.1 billion individuals considered overweight or obese worldwide [20]. These increases were seen in both developed and developing countries. These data have an important impact on public health. In fact, for every 5-unit increase in BMI above 25 kg/m^2^, the overall mortality increases by 29%, vascular mortality by 41%, and diabetes-related mortality by 210% [20]. Excess energy from the diet is stored as fat in the white adipose tissue, distributed widely throughout the body and affecting whole-body homeostasis through metabolic, endocrine, and immune functions.

Data from the literature indicated that obese individuals were more susceptible to respiratory virus infection, and to SARS-CoV-2 infection, and showed a greater severity of illness and a worse outcome after infection, including higher rates of hospitalization, admission to ICU, and death [20]. However, obese subjects more frequently have other chronic conditions associated with a severe outcome of COVID-19 (CVDs, diabetes, liver, or kidney disease, etc.) [21].

Our case–control study investigated the role of obesity on the outcome of patients with SARS-CoV-2 infection, comparing the outcome of obese patients with those with BMI < 30 kg/m^2^, excluding other confounding factors. In fact, excluding gender, the patients with and without obesity were pair matched for the most important factors associated with the severity of COVID-19: age, CCI, and number of co-pathologies. Thus, although the Cases and Controls had similar age, CCI, and number of co-pathologies, the obese patients appeared more compromised than those with normal BMI at admission, showing lower PaO_2_/FiO_2_ ratio (*p* = 0.014), a parameter correlated with the efficiency of the respiratory function. Moreover, and more interestingly, the outcome of the obese patients was more severe: a higher percentage of patients needing intensive care (*p* < 0.001) or who died during hospitalization (*p* = 0.046) was observed in the obese patients than in those with BMI < 30 kg/m^2^.

Several previous studies evaluated this subject and demonstrated the association between overweight and severe outcome of COVID-19 [12,22,23,24,25], but often without considering other confounding factors. A Dutch study showed that 90% of the SARS-CoV-2-infected patients with respiratory failure had BMI higher than 25 kg/m^2^ and that the severity of the disease increased significantly with a high BMI [26]. Concurrently, COVID-19 patients requiring mechanical ventilation in a Seattle-based cohort had a mean BMI of 33 kg/m^2^ [22]. A southern California–based study linked BMI > 40 to greater relative risk among patients aged 60 years or younger and in men [23]. An international multicenter retrospective cohort study revealed a linear association between BMI and the need for invasive mechanical ventilation for critically ill COVID-19 patients, which was more pronounced in younger females and independent of other metabolic risk factors. In addition, a significant association between BMI and mortality was observed in obesity class III (≥40 kg/m^2^) [24].

There are several factors that may explain the association between obesity and severe outcome of COVID-19.

Obesity is associated with insulin resistance and over-activity of the renin–angiotensin–aldosterone system, which is related to worse outcomes in COVID-19 infection. ACE2, used by SARS-CoV-2 to infect cells, is more expressed in adipose tissue than in the lung. The obese population has more adipose tissue and consequently higher ACE2 levels that may enable the entry of SARS-CoV-2 into adipocytes, making adipose tissue an important viral reservoir able to spread the virus to other organs. Moreover, obesity is the leading cause of diabetes, which is also causally linked to elevated ACE2 expression. Cells expressing ACE2 are also connected to the progression of idiopathic pulmonary fibrosis. Adipocytes may substantially contribute to the production of circulating angiotensinogen, which after metabolism by renin and ACE produces angiotensin II (Ang II). Liu et al. observed that patients with COVID-19 infection were shown to have elevated Ang II levels that correlated with the severity of lung injury [12]. High Angiotensin II levels in the lung can induce pulmonary vasoconstriction, which leads to ventilation/perfusion mismatch and hypoxemia, as well as inflammation and oxidative damage, promoting acute lung injury [12].

The literature highlights that PaO2/FiO2 ratio at admission is a predictor of worse CT score in COVID-19 patients and a negative outcome of the disease [17]. Considering the lower PaO2/FiO2 ratio in patients with obesity compared with patients without, we underline that obesity is associated with impaired pulmonary function, with decreased expiratory reserve volume, functional capacity, and respiratory system compliance. Increased abdominal obesity compromises pulmonary function in supine patients by decreased diaphragmatic excursion, while the base of the lung ventilation is also impaired, resulting in reduced oxygen-saturated blood levels [20]. Furthermore, chronic low-grade inflammation and increased levels of circulating pro-inflammatory cytokines associated with obesity, such as leptin, tumor necrosis factor a, and interleukin 6, may impair immune response and affect the lung parenchyma [20].

The analysis in the present study showed that patients with diabetes had more frequent arterial hypertension (61% vs. 49.6%, *p* = 0.027). A recent meta-analysis on the impact of arterial hypertension on worse outcome in COVID-19 patients revealed a non-independent role in mortality [25]. Arterial hypertension is one of the comorbidities that, in association with others, define a patient that more frequently could have the worst outcome. Our data showed a high prevalence of arterial hypertension in obese patients, in line with studies published before, [9] but considering the setting of patients included in our study and the comorbidity associated with patients with obesity, compared to patients without, it is not clear if arterial hypertension is a leading actor or a spectator.

These data explain why obesity is considered one of the risk factors for the progression of COVID-19 infection and evolution of respiratory insufficiency and suggest that all patients with BMI > 30 should be considered to receive antivirals or monoclonal antibodies for COVID.

Our study shows several limitations. Firstly, its retrospective design, and secondly, not all patients included in the initial cohort had BMI data, even though the demographic and clinical data were similar in the subjects for whom the datum of BMI was available and in those for whom it was not, and we do not have BMI classes. Thirdly, no data were available on vitamin D’s role. Lastly, due to the historical period of our study, our cohort did not include subjects who were vaccinated against SARS-CoV-2 and/or with early treatments (antivirals or monoclonal antibodies), nor the impact of different variants. On the other hand, the strengths of our study may be the pair-match design, number of subjects enrolled, and multicenter design.

In conclusion, in our pair-matched 1:2 real-life study, we confirmed an association between obesity and the severe outcome of patients with COVID-19. In addition, patients with obesity showed a higher prevalence of arterial hypertension and a lower PaO_2_ FiO2 ratio at admission, but the impact of arterial hypertension in COVID-19 progression is still misunderstood. Thus, considering the pharmacological baggage we currently have and considering the relevant pathogenicity of the Omicron variant, and the results of our work, the BMI ≥ 30 kg/m^2^ is still a very important comorbidity in COVID-19 patients determining worse outcomes. Therefore, early therapy for SARS-COV-2 should be evaluated, whether antiviral or monoclonal antibodies, to avoid the development of severe disease.

## Figures and Tables

**Table 1:** Demographic and clinical parameters in patients in the Case and Control groups.

	Case Group	Control Group	*p*-Value
No. of patients	141	282	
Males, No. (%)	95 (67.4)	190(67.4)	1 ^a^
Age, years, median (Q1–Q3)	58 (48–69)	58 (48–69)	0.925 ^b^
Charlson comorbidity index, median (Q1–Q3)	2 (1–4)	2 (1–4)	0.497 ^c^
Number of comorbidities for each patient, median (Q1–Q3)	1(1–2)	1(0–2)	0.197 ^c^
No. (%) of patients with hypertension	86 (61)	140 (49.6)	0.027 ^a^
No. (%) of patients with cardiovascular disease	34 (24.1)	90 (31.9)	0.097 ^a^
No. (%) of patients with diabetes	37 (26.2)	57 (20.2)	0.160 ^a^
No. (%) of patients with chronic obstructive pulmonary disease	22 (15.6)	27 (9.6)	0.068 ^a^
No. (%) of patients with chronic liver disease	6 (4.3)	8 (2.8)	0.438 ^a^
No. (%) of patients with chronic kidney disease	16(11.3)	22(7.8)	0.229 ^a^
No. (%) of patients with malignancy	5 (3.5)	13 (4.6)	0.609 ^a^
No. (%) of patients with dementia	2 (1.4)	13 (4.6)	0.095 ^a^

Footnotes: ^a^ Chi-square test; ^b^ Student’s t-test; ^c^ Mann–Whitney test.

**Table 2:** Clinical and laboratory parameters at admission in patients in the Case and Control groups.

	Case Group	Control Group	*p*-Value
No.of patients	141	282	
No. (%) of patients with fever	87 (62.6)	156 (55.5)	0.167 ^a^
No. (%) of patients with dyspnea	103 (74.6)	193 (68.7)	0.208 ^a^
No. (%) of patients with asthenia	23 (16.7)	79 (28.2)	0.01 ^a^
No. (%) of patients with cough	45 (32.6)	89 (31.8)	0.865 ^a^
No. (%) of patients with ageusia/dysgeusia	4 (2.9)	4 (1.4)	0.302 ^a^
No. (%) of patients with anosmia/hyposmia	3 (2.2)	6 (2.1)	0.984 ^a^
No. (%) of patients with diarrhea	10 (7.2)	8 (2.9)	0.038 ^a^
No. (%) of patients with skin lesions	0 (0)	0 (0)	-
Days from admission to discharge, median (Q1–Q3)	14 (10–20)	15 (10–21)	0.555 ^c^
No. (%) of patients with pneumonia	138 (97.9)	263 (93.6)	0.057 ^a^
Median (Q1–Q3) PaO2/FiO2 Ratio (P/F)	194 (119.5–300)	232 (156–329)	0.014 ^c^
Median (Q1–Q3) white blood cells	7900 (5425–10355)	7820 (5430–10730)	0.858 ^c^
Median (Q1–Q3) International Normalized Ratio (INR)	1.11 (1.04–1.19)	1.11 (1.04–1.21)	0.775 ^c^
Median (Q1–Q3) Blood creatinine	0.9 (0.8–1.1)	0.84 (0.7–1.025)	0.050 ^c^
Median (Q1–Q3) ALT	36 (26–49)	31 (21–51)	0.130 ^c^
Median (Q1–Q3) AST	38 (26–58)	33 (22–63.5)	0.230 ^c^
Median (Q1–Q3) total bilirubin	0.6 (0.5–0.8)	0.6 (0.41–0.85)	0.946 ^c^
Median (Q1–Q3) creatine phosphokinase (CPK) at admission	99 (54.5–298)	88 (57–166)	0.347 ^c^
Median (Q1–Q3) lacticodehydrogenase (LDH)	323 (254–419)	288 (229–411)	0.099 ^c^

Footnotes: ^a^ Chi-square test; ^c^ Mann–Whitney test.

**Table 3:** Clinical outcome of patients in the Case and Control groups.

	Case Group	Control Group	*p*-Value
No.of patients	141	282	
No. (%) of patients with mild or moderate outcome	65(46.1)	189(67)	0.0001 ^a^
No. (%) of patients needing intensive care treatment	59 (41.8)	75 (26.6)	0.001 ^a^
No. (%) of patients who died during hospitalization	17 (12.1)	18 (6.4)	0.046 ^a^

Footnotes: ^a^ Chi-square test.

## Data Availability

The data that support the findings of this study are available from the corresponding author, N.C., upon reasonable request.

## Supplementary Materials

The following supporting information can be downloaded at: https://www.mdpi.com/article/10.3390/jcm12124055/s1, Table S1: STROBE Checklist.
