# Supplementary material for: Obesity as a Risk Factor of Severe Outcome of COVID-19: A Pair-Matched 1:2 Case–Control Study
Source: J Clin Med. 2023 Jun 15;12(12):4055. doi: 10.3390/jcm12124055 (PMC10298877; doi:10.3390/jcm12124055)
Supplement: Supplementary file 1 [file jcm-12-04055-s001.zip › jcm-2387522-supplementary.pdf]

OBESITY AS RISK FACTOR OF SEVERE OUTCOME OF COVID-19: A CASE-CONTROL STUDY

Antonio Russo[1], Mariantonietta Pisaturo[1], Verdiana Zollo[1], Salvatore Martini[1], Paolo Maggi[2], Fabio Giuliano Numis[3], Ivan Gentile[4], Vincenzo Sangiovanni[5], Vincenzo Esposito[6], Vincenzo Bianco[7], Giosuele Calabria[8], Raffaella Pisapia[9], Angelo Salomone Megna[10], Alfonso Masullo[11], Elio Manzillo[12], Grazia Russo[13], Roberto Parrella[14], Giuseppina Dell'Aquila[15], Michele Gambardella[16], Antonio Ponticiello[17], Lorenzo Onorato[1], Nicola Coppola[1] on behalf of CoviCam group

1. Infectious Diseases Unit, Department of Mental Health and Public Medicine, University of Campania "L. Vanvitelli", Napoli, Italy
2. Infectious Diseases Unit, A.O. S Anna e S Sebastiano Caserta, Italy
3. Emergency Unit, PO Santa Maria delle Grazie, Pozzuoli, Italy
4. Infectious Diseases Unit; University Federico II, Naples, Italy
5. Third Infectious Diseases Unit, AORN dei Colli, P.O. Cotugno, Naples, Italy
6. IV Infectious Diseases Unit, AORN dei Coli, PO Cotugno, Naples, Italy
7. Hepatic Infectious Diseases Unit, AORN dei Colli, PO Cotugno, Naples, Italy
8. IX Infectious Diseases Unit, AORN dei Coli, PO Cotugno, Naples, Italy
9. First Infectious Diseases Unit, AORN dei Coli, PO Cotugno, Naples, Italy
10. Infectious Diseases Unit, A.O. San Pio, PO Rummo, Benevento, Italy
11. Infectious Diseases Unit, A.O. San Giovanni di Dio e Ruggi D'Aragona Salerno, Italy
12. VIII Infectious Diseases Unit, AORN dei Coli, PO Cotugno, Naples, Italy

13. Infectious Diseases Unit, Ospedale Maria S.S. Addolorata di Eboli, ASL Salerno, Italy

14. Respiratory Infectious Diseases Unit, AORN dei Colli, PO Cotugno, Naples, Italy

15. Infectious Diseases Unit, AO Avellino, Italy

16. Infectious Diseases Unit, PO S. Luca, Vallo della Lucania, ASL Salerno, Italy

17. Pneumology Unit, AORN Caserta, Italy

SUPPLEMENTARY DATA

Supplementary Table S1: STROBE Cecklist

	Item No.	Recommendation	Page No.
**Title and abstract**	1	(*a*) Indicate the study's design with a commonly used term in the title or the abstract	1
		(*b*) Provide in the abstract an informative and balanced summary of what was done and what was found	3
**Introduction**			
Background/rationale	2	Explain the scientific background and rationale for the investigation being reported	4
Objectives	3	State specific objectives, including any prespecified hypotheses	5
**Methods**			
Study design	4	Present key elements of study design early in the paper	5
Setting	5	Describe the setting, locations, and relevant dates, including periods of recruitment, exposure, follow-up, and data collection	5
Participants	6	*Case-control study*—Give the eligibility criteria, and the sources and methods of case ascertainment and control selection. Give the rationale for the choice of cases and controls	5

		Case-control study—For matched studies, give matching criteria and the number of controls per case	5
Variables	7	Clearly define all outcomes, exposures, predictors, potential confounders, and effect modifiers. Give diagnostic criteria, if applicable	6
Data sources/ measurement	8*	For each variable of interest, give sources of data and details of methods of assessment (measurement). Describe comparability of assessment methods if there is more than one group	*Supplemeantary data*
Bias	9	Describe any efforts to address potential sources of bias	5
Study size	10	Explain how the study size was arrived at	6
Quantitative variables	11	Explain how quantitative variables were handled in the analyses. If applicable, describe which groupings were chosen and why	Table 1
Statistical methods	12	(*a*) Describe all statistical methods, including those used to control for confounding	6
		(*b*) Describe any methods used to examine subgroups and interactions	6
		(*c*) Explain how missing data were addressed	6
		Case-control study—If applicable, explain how matching of cases and controls was addressed	6
		(*e*) Describe any sensitivity analyses	6
**Results**			
Participants	13*	(a) Report numbers of individuals at each stage of study—eg numbers potentially eligible, examined for eligibility, confirmed eligible, included in the study, completing follow-up, and analysed	Figure 1, page 7
		(b) Give reasons for non-participation at each stage	Figure 1
		(c) Consider use of a flow diagram	Figure 1

Descriptive data	14*	(a) Give characteristics of study participants (eg demographic, clinical, social) and information on exposures and potential confounders	7
		(b) Indicate number of participants with missing data for each variable of interest	Supplementary data
Outcome data	15*	*Case-control study*—Report numbers in each exposure category, or summary measures of exposure	*Table 2*
Main results	16	(*a*) Give unadjusted estimates and, if applicable, confounder-adjusted estimates and their precision (eg, 95% confidence interval). Make clear which confounders were adjusted for and why they were included	Not applicable
		(*b*) Report category boundaries when continuous variables were categorized	7, table 2
		(*c*) If relevant, consider translating estimates of relative risk into absolute risk for a meaningful time period	Not applicable
Other analyses	17	Report other analyses done—eg analyses of subgroups and interactions, and sensitivity analyses	Not applicable
**Discussion**			
Key results	18	Summarise key results with reference to study objectives	9
Limitations	19	Discuss limitations of the study, taking into account sources of potential bias or imprecision. Discuss both direction and magnitude of any potential bias	10
Interpretation	20	Give a cautious overall interpretation of results considering objectives, limitations, multiplicity of analyses, results from similar studies, and other relevant evidence	10
Generalisability	21	Discuss the generalisability (external validity) of the study results	9-10
**Other information**			

Funding	22	Give the source of funding and the role of the funders for the present study and, if applicable, for the original study on which the present article is based	12
